# The role of anterior segment optical coherence tomography in post-cataract surgery Descemet membrane detachment

**DOI:** 10.1007/s10792-024-03397-y

**Published:** 2025-02-18

**Authors:** Francesco Ruggeri, Daria Rullo, Elisa Maugliani, Nicola Trotta, Chiara Ciancimino, Mariachiara Di Pippo, Fabio Guglielmelli, Solmaz Abdolrahimzadeh

**Affiliations:** 1 Opthalmology Unit, Neurosciences, Mental Health, and Sense Organs (NESMOS) Department, Faculty of Medicine and Psychology, Sapienza University of Rome, Via di Grottarossa 1035/1039, 00189 Rome, Italy; 2 St. Andrea Hospital, Via di Grottarossa 1035/1039, 00189 Rome, Italy

**Keywords:** Anterior segment optical coherence tomography, AS-OCT, Corneal edema, Descemet’s membrane detachment, Cataract surgery, Phacoemulsification, Pneumodescemetopexy

## Abstract

This review seeks to evaluate anterior segment optical coherence tomography (AS-OCT) in the diagnostic procedure and management of Descemet’s membrane detachment (DMD) in cataract surgery. DMD may present diagnostic challenges, particularly in pronounced corneal edema where traditional methods such as slit lamp biomicroscopy may be inadequate in evaluating the corneal layers. The role of AS-OCT in providing high-resolution images in the preoperative, intraoperative, and postoperative phases of cataract surgery is analyzed with a focus on its role in the early diagnosis of DMD and in evaluating the extent, morphology, and topographic localization of DMD allowing for immediate intervention during surgery and precise pneumodescemetopexy procedures where conservative treatment has failed. This review explores the integration of AS-OCT into the standard perioperative diagnostic workflow, highlighting its potential role in the prevention, accurate diagnosis, and prompt management of DMD, a complication of cataract surgery that, while low in incidence, can be highly disruptive when it occurs. The emerging role of artificial intelligence (AI) in AS-OCT analysis of anterior segment conditions and surgical procedures is discussed, though refinement of AI algorithms is warranted.

## Introduction

Descemet’s membrane (DM) integrity is crucial to preserve the optical transparency of the cornea. The corneal structure comprises several layers: the epithelium, Bowman’s membrane, the stromal layer, DM, and the endothelial lining [1]. Within these layers three cellular subtypes can be identified: surface epithelial cells, keratocytes within the stromal layer, and endothelial cells on the innermost surface each supported by a matrix of collagen and glycosaminoglycans [1]. A rupture within DM may permit fluid infiltration into the gap separating the corneal stroma from DM, resulting in a Descemet’s membrane detachment (DMD), a condition that leads to stromal edema, epithelial bullae formation, and a profound decline in visual acuity. Frequently spontaneous DM reattachment occurs, and no intervention is needed, but more severe cases could potentially lead to endothelial decompensation if left untreated [2–7].

DMD mainly arises following various intraocular procedures, with a significant proportion resulting from cataract extraction, with a reported 2.5% following extracapsular extraction and 0.044–0.5% following phacoemulsification [8–12].

Conventional slit lamp biomicroscopy conducted by an experienced clinician is effective in detecting DMD in cases of relatively clear corneas [12]. However, its diagnostic accuracy diminishes significantly in extensive corneal edema, a condition that often accompanies DMD, which can make diagnosis solely through slit lamp examination challenging, often not offering sufficient detail to fully evaluate the morphology and precise location of the detachment, which are important in the treatment choice. [13, 14].

Optical coherence tomography (OCT), since its introduction in 1991 by Huang and colleagues [15], has seen vast diffusion and is now a standard examination for diseases of the posterior pole. This ground-breaking technology has significantly improved our understanding of pathological mechanisms in retinal and choroidal disease and has substantially upgraded the quality of patient care [16, 17]. The extension of this technology to anterior segment optical coherence tomography (AS-OCT) has brought notable improvements to ophthalmic imaging by enabling non-invasive visualization of anterior segment structures with enhanced precision at a microscopic level [18–21]. Swept-source (SS) technology, incorporated into modern biometers, has furthered improved imaging. [22] Like all innovative technologies, AS-OCT has enabled the acquisition of valuable novel data that enhance our understanding of previously overlooked pathological processes. Initially adopted in research and specialized tertiary hospitals, such technologies gradually make their way into more routine medical practice.

This review aims to analyze data from existing case reports, case series, and original articles to highlight the contributions of AS-OCT to perioperative evaluation in cataract surgery, especially in predicting, comprehending, and treating DMD. The potential integration of AS-OCT in standard post operative diagnostic workflow is discussed with an analysis of the current limitations on the use of perioperative AS-OCT in routine cataract surgery. Furthermore, the emerging role of artificial intelligence (AI) in AS-OCT analysis is explored, shedding light on its potential to further enhance perioperative diagnostics and management in cataract surgery.

## Descemet’s membrane detachment etiology and classification

DMD results from a gap separating the corneal stroma from DM mainly following various intraocular procedures. DMD is reported after refractive surgery, keratoplasty and cataract surgery [2, 4, 5, 12]. Post-cataract surgery DMD is typically linked to factors such as clear corneal incision, repeated misalignment during surgical instrument insertion, accidental placement of surgical instruments or viscoelastic material between DM and the stromal layer, or incorrect handling of irrigation/aspiration equipment [23]. Approximately 47% of cataract surgeries that appear uncomplicated may still involve cases of DMD that are not readily apparent. [19, 24, 25]. DMD can result in persistent post-surgical corneal edema, potentially causing severe endothelial decompensation and compromising visual acuity, thus, early diagnosis is critical in the sensitive postoperative recovery phase [4–6].

**Fig. 1:**
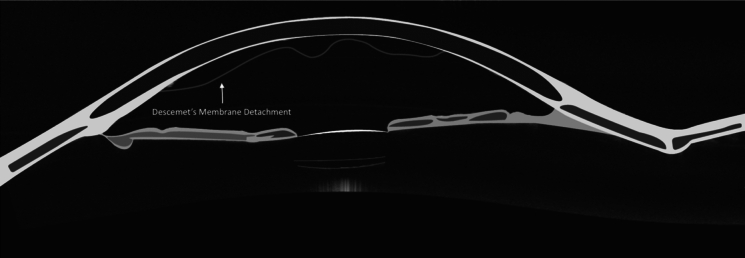
Schematic illustration of anterior segmet optical coherence tomography. Hyperreflective line showing Descemet’s membrane detachment—arrow (graphic illustration courtesy of Dariush Rahimi)

DMD can also manifest spontaneously in pellucid marginal corneal degeneration and keratoconus [2, 3, 26]. Moreover, it can be instigated by an array of infrequent triggers, including caustication from chemical agents, hemorrhage, deep abscesses, DM contraction within areas of peripheral anterior synechiae, and in phthisical eyes [8–11, 27]. Historical insights on DMD were provided by Samuels in 1928, who classified active and passive forms. Active DMD involves exudates, blood, foreign bodies, or tumors exerting force on DM, while passive DMD is related to contracted and detached DM in inflamed eyes. However, these forms were deemed more pathologically significant than surgically relevant [11, 27]. A subsequent classification by Mackool and Holtz in 1977 introduced distinctions based on the extent of post-operative DMD. The authors reported that planar DMD, characterized by DM separations of less than 1 mm, often experienced spontaneous reattachment, while non-planar DMD, featuring separations exceeding 1 mm, typically required surgical intervention. Both the planar and non-planar DMD categories were further distinguished into two distinct subdivisions: (1) peripheral DMD, with detachment within the 3 mm of the periphery, or (2) combined central and peripheral DMD with additional involvement of the central corneal area [9]. A graphical illustration of DMD is presented in Fig. 1.

Liu Zuguo and colleagues, in 1990, proposed a new classification where DMD was classified in five stages based on detachment extension: less than 1/8 of the corneal area defined as local; between 1/8 and 1/4 of the corneal area defined as mild; between 1/4 and 1/2  of the corneal area defined as moderate; equal to or greater than 1/2 of the corneal area defined as severe; and complete detachment defined as full [18, 28]. This classification method was based only on an estimation of the area of corneal edema and the range of DMD due to diagnosis limited to slit lamp examination without imaging methods [18, 28].

The subsequent development of AS-OCT enabled a higher diagnostic accuracy and accurate localization and quantification of DMD [20, 27, 29, 30]. AS-OCT was found to have a 36% better diagnostic accuracy over conventional slit lamp microscopy, gonioscopy, and Schiemflug imaging for hazy corneas [31, 32]. Classification systems introduced by Jacob and colleagues (2015) and Guo and colleagues (2018) based on AS-OCT findings enabled a more nuanced categorization of DMD severity, aiding in treatment planning and prognostication [18, 27]. Jacob and colleagues presented an innovative classification framework for DMD constructed through an exhaustive analysis of AS-OCT data, taking into consideration factors such as etiopathogenesis, clinical presentation, and intra-operative features, [27]. This categorization distinguished DMD in several forms, each with distinct characteristics and implications. Rhegmatogenous Descemet’s detachment involved DM separation due to a tear or hole at Schwalbe’s line where AS-OCT showed an undulating hyperreflective line within the anterior chamber (AC) [27]. Tractional Descemet’s detachment, though rare, emerged from the DM being pulled or shortened due to underlying inflammation or fibrosis, or complications at surgical junctions. AS-OCT showed a tense and straightened appearance, stretching between the eye attachment sites [27]. Bullous Descemet’s detachment was observed when the DM bulged into the AC, typically without any visible breaks or occasionally with a very small puncture. This bulging could arise from the injection of various substances like viscoelastic or blood into the space above DM during specific eye procedures, such as viscocanalostomy or cataract surgery, creating a convex hyperreflective signal on AS-OCT [27]. Complex Descemet’s detachment included a range of DM alterations, including macrofolds, rolls, and scrolled edges, where AS-OCT revealed the detailed patterns and adhesions in AC. Each of these conditions required careful evaluation using AS-OCT for appropriate diagnosis and subsequent management [18, 27, 29, 30, 33].

## AS-OCT characteristics

AS-OCT software produces tomographic images that are two- or three-dimensional through the measurement of the time delay of light echoed back from various ocular tissues. This technology, an advancement in OCT imaging, specifically targets the anterior segment of the eye [34]. AS-OCT has proven to be a vital instrument for diagnosing and evaluating the progression of anterior segment pathologies including outcomes of surgical sequalae with applications in both clinical practice and research [35]. AS-OCT systems are categorized based on the light source wavelengths. Some employ a wavelength of 1310 nm, while others convert from a retinal scanner using 830 nm [34, 36]. For instance, time-domain OCT (TD-OCT) systems, such as the Visante OCT (Carl Zeiss Meditec, AG, Jena, Germany) and Heidelberg slit lamp OCT (Heidelberg Engineering, Heidelberg, Germany), utilize a 1310 nm wavelength. These systems offer lower A-scan rates and sensitivity compared to Fourier Domain OCT (FD-OCT) systems [36].

Spectral domain (SD-OCT) and SS-OCT are two branches of the FD-OCT, each with distinct characteristics. Devices like the Spectralis (Heidelberg Engineering, Heidelberg, Germany) and RTVue XR (Optovue Inc., Fremont, CA, USA) use SD-OCT with high-speed acquisition using different wavelengths [36, 37]. The latest in AS-OCT technology is SS-OCT, exemplified by the Cassia SS-OCT (Heidelberg Engineering, Heidelberg, Germany) and ANTERION (Heidelberg Engineering, Heidelberg, Germany), which utilizes a high-speed 1310 nm SS laser. This technology allows for comprehensive visualization of anterior segment structures including the ciliary body and lens thickness [23, 36]. While newer technologies are advancing, TD-OCT remains relevant, especially for corneal applications. Its longer wavelength reduces scattering, facilitating better penetration through corneal or scleral opacities and offering enhanced imaging compared to FD-OCT, despite the latter’s higher resolution [37].

In AS-OCT images, the corneal structure is delineated into distinct layers with unique optical properties, the outermost hyper-reflective line, typically less than 5 µm, is indicative of the tear film; the underlying hypo-reflective zone corresponds to the corneal epithelium(50 and 70 µm); Bowman’s layer is visualized as a discrete linear formation exhibiting similar reflectivity to the stroma; the stromal layer displays a range of variable reflectivity (approximately 500 µm); the combined region of the pre-Descemet’s layer and the DM manifests as a prominently hyper-reflective band [38, 39]. AS-OCT is valuable in cataract surgery, from preoperative assessment to postoperative care to calculating intraocular lens (IOL) power providing comprehensive corneal measurements and detailed visualization of DM and IOL stability [35, 40–42]. Furthermore, AS-OCT is a non-invasive approach valuable in evaluating a wide range of eye conditions [35, 43]. It is instrumental in detecting and monitoring conjunctival diseases like pterygium and pinguecula, evaluating conjunctival and scleral thickness for diagnosing inflammatory diseases, tumors, scleritis, and in assessing corneal conditions such as keratoconus, dystrophies, and keratitis. AS-OCT enables to measure corneal thickness that is vital for refractive surgery eligibility, and corneal ectasia management. AS-OCT is also used for the AC angle and depth assessment, crucial for diagnosing conditions like narrow-angle glaucoma, and iris and lens evaluations especially using the SS systems [23, 36, 44, 45]. Additionally, it is used in the management of dry eye syndrome by examining the meniscus and the tear film, and in assessing pupil diameter and reactivity [34, 35, 41, 42] (Table 1).

**Table 1:** Main characteristics of AS-OCT devices

AS-OCT domain		Devices	A-scan rate (scan/s)	Optical source	Wavelength	Pros & Cons
TD-OCT		Visante OCT (Carl Zeiss Meditec, Jena, Germany)	2000 [39, 46]	Superluminescent diode [39]	1310 nm [39, 46]	Lower sensitivity [36]
		Heidelberg slit lamp OCT (Heidelberg Engineering, Heidelberg, Germany)	200 [27, 46]			Reduced scattering [37]
						Better penetration through corneal opacity [37]
FD-OCT	SD-OCT	Spectralis (Heidelberg Engineering, Heidelberg, Germany)	40,000 [39, 46]	Superluminescent diode [46]	820 nm ± 20 nm [39, 46]	High resolution [23, 36]
		RTVue XR (Optovue Inc., Fremont, CA, USA)	70,000 [47]			Standard penetration [23, 36]
		iVue80 (Optovue, Fremont, CA, USA)	80,000 [39, 48]			
		Cirrus OCT (Carl Zeiss Meditec, Jena, Germany)	27,000 [39, 46]			
		Topcon 3D OCT 1000 Mark II,Tokyo, Japan)	18,000 [9]			
	SS-OCT	Casia SS-1000 OCT (Tomey, Nagoya, Japan)	30,000 [39, 46]	Swept-source laser [46]	1310 nm ± 10 nm [39, 46, 49]	Higher resolution
		Anterion OCT (Heidelberg Engineering, Heidelberg, Germany)	16,640 [49]			Excellent penetration [23, 36]
		Triton OCT (Topcon Corporation, Tokyo, Japan)	100,000 [39, 46]			Comprehensive visualization of all anterior segment [23, 36]

Time-domain Optical coherence tomography (TD-OCT); Spectral domain Optical coherence tomography (SD-OCT); Swept source Optical coherence tomography (SS-OCT); Fourier domain Optical coherence tomography (FD-OCT); Anterior segment Optical coherence tomography (AS-OCT)

## Anterior segment optical coherence tomography in Descemet’s membrane detachment

### Preoperative risk factors

AS-OCT evaluation of the preoperative factors associated with DMD is not common clinical practice. Nevertheless, a thorough analysis of the existing literature on postoperative DMD reveals several critical predisposing conditions of the anterior segment that might contribute to this complication. A shallow AC is reported as being a potential risk factor for postoperative DMD together with hazy cornea and hypotony [11, 21, 42]. Several cases of cataract surgery later presenting postoperative DMD revealed a shallow AC [21, 50]. In three out of 14 patients in the study by Wylęgała and colleagues, a history of primary angle closure glaucoma was found [20, 50], suggesting a possible correlation between this anatomical presentation and a higher risk for DMD occurrence. AS-OCT offers a quantifiable and objective measurement of the anterior chamber depth (ACD), calculated by measuring the straight-line distance from the corneal endothelium to the anterior surface of the lens [51]. Furthermore, AS-OCT has demonstrated reliability in determining the AC volume, derived from the area of the AC through computational methods [52, 53]. In this context AS-OCT could be useful in facilitating a more informed surgical plan that accounts for individual patient anatomy and ocular condition [50]. Optical biometry can provide parameters such as axial length, ACD, and keratometry particularly with the advent of novel technology in biometry devices that utilize SS-OCT [54].

The role of cataract characteristics in potentially influencing the risk of DMD in phacoemulsification surgery has been evaluated. Sharma and colleagues [19] and Kumar and colleagues [50] indicated no significant differences in their respective studies in postoperative DMD occurrence related to the degree of cataract severity. However, a higher cataract density is suggested as a possible predisposing factor for post cataract surgery DMD in the single case reported by Agraval and colleagues [29].

Corneal endothelial changes may have implications in predisposing to DMD, with Fuchs endothelial corneal dystrophy (FECD) being reported as a potential risk factor [55, 56]. In four of the 16 eyes with DMD reported by Ti and colleagues [56] endothelial disease or presumed endothelial disease was mentioned, with FECD diagnosis reported in three eyes and significant guttate and pleomorphism reported in one eye. Kumar and colleagues reported two cases of DMD in a patient with FECD on a total of 161 eyes examined [50]. AS-OCT is effective in evaluating corneal morphology, notably in patients with corneal dystrophies, facilitating detailed analysis of the location and nature of abnormalities within the corneal layers [57, 58]. In FECD AS-OCT images showed a thickened DM which appears as a hypo-reflective band with numerous nodular protrusions from the endothelial cells [59]. Moreover, the corneal structure often exhibited edema and epithelium bullae formation, indicative of advanced disease state [57, 59–61]. Yasukura and colleagues proposed a new grading system for FECD in 2021 [62] with the aim of translating the modified Krachmer grade, utilized in FECD slit lamp evaluation, towards an AS-OCT approach, more objective and reproducible. The modified Krachmer classification grades FECD from 1 to 5 (without corneal edema) and 6 (with corneal edema). Yasukura and colleagues introduced a three-tiered classification through AS-OCT evaluation [63]. Grade 1 was identified by the presence of guttae with a corresponding elevation in the central corneal posterior map. Grade 2 involved stromal edema indicated by a depression in the central cornea on the posterior elevation map. Grade 3 was characterized by both epithelial and stromal edema, discernible by a central corneal thickness exceeding 700 µm within a 3-mm radius as determined by AS-OCT corneal pachymetry [62].

Preoperative clinical examination with the slit lamp and AS-OCT evaluation reveals the multifactorial nature of postoperative DMD risk, showing the role of anatomical variations and corneal and lens conditions in predisposing to this complication. Nevertheless, while AS-OCT enables precise measurement and assessment of ocular structures and certain preoperative anatomical characteristics, its prognostic value in preoperatively predicting DMD has not been found to be significant. The utility of AS-OCT for detailed imaging of anterior segment structures and lens density is evident; however, the integration of this technology into standard preoperative evaluation for cataract patients does not appear to be justified solely for predicting DMD, a condition which remains rare following phacoemulsification [8–12]. Preoperative evaluation in cataract surgery through AS-OCT is a valuable component within specialized tertiary hospitals and research institutions with the potential to enhance our understanding of DMD and inform future advancements.

### Intraoperative risk factors

The intraoperative factors contributing to DMD are diverse, encompassing both technique-specific and incident-related conditions. Identified surgical risk factors for DMD can be categorized into three main groups: incisional, instrumental, and operator-related [63]. Incisional risk factors include the employment of dull surgical blades [64–67], incisions that are improperly angled or too shallow [68], main incisions that are too narrow for the phacoemulsification probe [42], or IOL insertion [69]. Operator-related factors involve interactions with the DM during irrigation/aspiration, inadvertent injections of antibiotics or viscoelastic beneath the stroma [70], and the experience level of the surgeon [56]. Xia and colleagues reported that the main cause of DMD is the insertion of a surgical instrument, particularly one that is not sharp, into the AC under low intraocular pressure (IOP) [33]. Several cases of DMD are reported after uneventful cataract surgeries, suggesting that even in the absence of complications DMD can occur [21, 31, 42].

The incision site is commonly part of the detachment as Sharma and colleagues noted in their study where postoperative AS-OCT confirmed the involvement of the main incision site in DMD in fourteen of the 43 eyes with postoperative DMD [19]. Similarly, Kumar and colleagues documented with AS-OCT that the surgical access site was implicated in forty-one of 161 eyes with DMD [50]. AS-OCT has been used to evaluate the methodology of the surgical incision, as opposed to the location, as a variable in DMD occurrence during cataract surgery. In the research conducted by Gharaee and colleagues, AS-OCT was systematically utilized during cataract surgery management to investigate the outcomes of various incision techniques, wound gape (epithelial gaping manifests as a fissure along the limbal verge of the external incision, while endothelial separation appears as a discontinuity or misalignment along the boundary of the internal incision), and stromal hydration methods practiced by different surgeons [71]. The goal was to discern whether certain methods held higher risks or led to a greater incidence of complications as compared to others. Using the built-in caliper of the FD-OCT device, the authors meticulously evaluated the clear corneal incisions by measuring the mean length and angle [72]. The measurement of the incision extent was determined by joining the points where the incision begins internally and terminates externally. Corneal thickness measurements were taken both at the point where the incision began at the corneal surface and where it entered the AC. Additionally, the authors assessed the angle of the incision by plotting a line that ran tangentially along the surface of the corneal epithelium, extending to intersect with a line joining the incision’s beginning and end points. The authors also measured the area above the incision, defined by incision curvature and the corneal surface, and the area below the incision, enclosed by the incision curve and the internal corneal surface [72]. When DMD occurred, it appeared as a hyper-reflective line in the internal wound surface [65, 72, 73]. However, the investigation found no meaningful link between the average angle of the primary incision and the occurrence of DMD [71]. Furthermore, no significant relationship was found between the number of planes in the surgical incision and the occurrence of DMD (*P* = 0.200) [71]. Similarly, the authors indicated no substantial link between DMD and the incision length/ angle (*P* = 0.520 and *P* = 0.600, respectively) [71]. However, the authors reported a DMD occurrence rate of 63% and 25%, respectively, in eyes where stromal hydration was performed at the main incision site and the eyes where it was not.

Xia and colleagues reported on 60 eyes undergoing phacoemulsification where high-speed AS-OCT (Visante OCT; Zeiss Meditec, Inc., Dublin, CA, USA) was performed a day before and after surgery. The authors found that AS-OCT could effectively identify early postoperative changes in the incision architecture such as wound stretching or gaping and DMD with remarkable detail [33]. They determined that the placement of the surgical incision, specifically the gap between the incision endothelial edge and the scleral spur, did not correlate with the incidence of DMD. Additionally, consistent with observations by previous authors [74], DMD showed no link to corneal endothelial cell count [33]. Chen and colleagues observed a solitary instance of DMD associated with a biplanar incision in cataract surgery with the femtosecond laser [63]. They noted that during a triplanar incision, the force exerted by surgical instruments during the irrigation/aspiration phase impacted both the endothelium and a portion of the stroma more evenly. In contrast, with a biplanar incision, the force was primarily concentrated on the endothelium. Based on their findings, the authors suggested that this concentration of force could lead to DMD in the presence of even a small tear and proposed that a triplanar incision might lessen the risk of DMD [63].

Inadvertent balanced saline solution (BSS) injection between DM and the stroma during hydration of the surgical wound was considered as another potential trigger for DMD. Agraval and colleagues documented a postoperative case of DMD, suggesting that it could have occurred during the hydration of the surgical wound following phacoemulsification [29]. Although AS-OCT was not used during the surgery, a subsequent postoperative application of the technology confirmed the presence of paracentral DMD [20]. However, in the research conducted by Gharaee and colleagues, intraoperative AS-OCT was methodically utilized to observe the impact of stromal hydration on phacoemulsification outcomes but the authors did not find correlation of stromal hydration with DMD (*P* = 0.420) [71].

The studies discussed illustrate that using AS-OCT intraoperatively can be highly beneficial at various stages of cataract surgery. For instance, Sharma and colleagues [19] and Kumar and colleagues [50] presented evidence of postoperative DMD originating from the main incision site during the initial steps of surgery, supporting the hypothesis that, with real-time feedback from intraoperative AS-OCT, the surgeon could detect such issues early, adjust the incision technique, and, most importantly, manage them intraoperatively. Additionally, Xia and colleagues [33] identified the use of dull-bladed instruments in the AC alongside low IOP as a significant risk factor. Real-time feedback from AS-OCT can assist the surgeon in adjusting their technique and making real-time modifications based on what is observed, potentially reducing complications.

The final steps of cataract surgery are crucial phases for the subsequent development of DMD and could, therefore, thoroughly benefit from the implication of intraoperative AS-OCT. Agraval and colleagues, in their case report [20], suggested that DMD might have resulted from the hydration of the suture at surgery conclusion. This situation presents an ideal case for the use of intraoperative AS-OCT, as it could allow the surgeon to detect and address the issue immediately by injecting an air bubble into the AC. This proactive step could benefit both the patient and surgeon to achieve rapid recovery and limit the costs and time associated with managing a postoperative complication.

Additional support for the intraoperative use of AS-OCT in cataract surgery is provided by the study conducted by Gharaee and colleagues. Although the authors reported no significant correlation between DMD occurrence and incision technique, they observed a significant difference in DMD incidence depending on whether stromal hydration was performed. This finding suggests that AS-OCT could assist in assessing and adjusting the stromal hydration technique or in closely monitoring this phase to help prevent DMD [71]. The authors [71], also stated that a more integrated and detailed assessment with AS-OCT could help determine the optimal incisional shape.

Although extensive evidence is still lacking, intraoperative AS-OCT in cataract surgeries shows significant potential for the immediate detection and management of DMD, enabling corrective actions during the procedure itself. Early intervention facilitated by AS-OCT may minimize the need for postoperative corrections, ensuring precise identification and timely treatment of DMD. While not yet widely available, intraoperative AS-OCT, where accessible, serves as a valuable tool in reducing postoperative DMD risk and enhancing surgical outcomes.

### Postoperative diagnosis and treatment

Following cataract surgery, under clear corneal conditions, an experienced ophthalmologist should be able to recognize the presence of DMD through slit lamp biomicroscopy [7]. However, the occurrence of unobvious DMD, even in cases where cataract surgery appears uncomplicated, is estimated to be around 47% [19, 75, 76]. AS-OCT is a crucial component in DMD diagnosis in cases where the AC is not clearly visualized, such as in severe corneal edema. It enables clear visualization of AC structures and discriminates between the corneal layers with almost histological detail. [14, 33, 39, 77]. In the two cases reported by Kothari and colleagues slit lamp examination failed to visualize the anterior segment due to severe corneal edema and in both cases AS-OCT (performed on postoperative day 5 and 8) was necessary in order to detect DMD and successfully treat the condition [78]. Guo and colleagues highlighted a case where AS-OCT was essential in accurately delineating the extent of DMD, an area where the slit lamp biomicroscopy fell short [18]. Similarly, AS-OCT was instrumental for diagnosis in a case presented by Xie and colleagues, where corneal edema pesisted and DMD was undetected by slit lamp examination for two months [23]. In situations of significant corneal edema, ultrasound biomicroscopy (UBM) is also an additional valuable imaging method for examining the AC. Nevertheless, AS-OCT has distinct benefits: it provides quicker image acquisition, does not require direct contact with the cornea, and allows for imaging of patients in a seated position [79, 80].

In the management of DMD, a variety of treatment modalities have been documented, although detachment could resolve spontaneously, especially in well confined cases. The treatment options reported in the literature fall into two main categories: a conservative method or a surgical approach. The first treatment option that usually proves to be successful in resolving most DMD cases is conservative treatment involving a topical administration regimen of corticosteroids or nonsteroidal anti-inflammatory drugs (NSAIDS), topical hyperosmotic agents, together with antibiotic coverage [19, 23, 29, 50, 70, 81].

Pneumodescemetopexy represents the second option for DMD treatment and it is utilized primarly or after a failed attempt with conservative treatment. Pneumodescemetopexy entails the injection of air or gas via a 27 or 30 gauge needle directly into the AC through a paracentesis to ensure that the volume created by the bubble allows the detached DM to remain adhered to the overlying stroma. [15] To further enhance the adherence of the DM, the fluid between the DM and the stroma can be drained, either through venting incisions or by aspiration using a needle. [42, 82].

The two main types of gas used are 20% sulfur hexafluoride (SF6) or 14% octafluoropropane (C3F8) [82]. Other surgical procedures include mechanical tamponade using viscoelastic injected into the AC although generally this method is used after pneumodescemetopexy failure [83, 84]. Intraocular pressure could increase when viscoelastic injection is performed so the use of topical or oral ocular pressure reducing drugs is suggested [13]. Another method reported in the literature is fixation of the DM to the stroma using 10–0 nylon transcorneal suture; this technique can also be combined with pneumodescemetopexy for possible persistent areas of DMD. [13, 85]. For cases of DMD unresponsive to the previously mentioned approaches, the final option remains keratoplasty, which can be a lamellar like Descemet membrane endothelial keratoplasty (DMEK) [13] and Descemet-stripping automated endothelial keratoplasty (DSAEK) [86] or a penetrating keratoplasty (PK) [87].

**Fig. 2:**
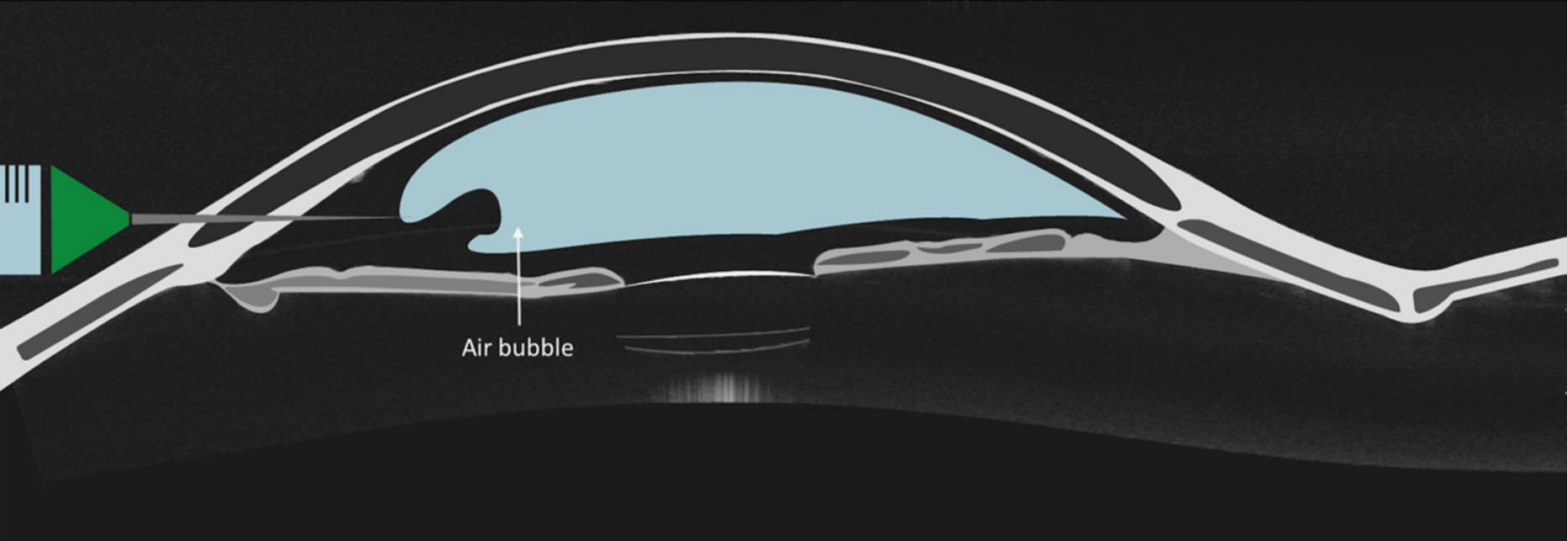
Schematic illustration of injection of air in the anterior chamber (graphic illustration courtesy of Dariush Rahimi)

Sharma and colleagues [19] used AS-OCT to categorize DMD, identifying planar DMD in 43 patients, involving less than one quadrant of the cornea. Based on this assessment, the authors opted to not administer any treatment and reported spontaneous resolution in all cases [19]. Kumar and colleagues reported on 161 cases of DMD examined using AS-OCT analysis tool to gauge the vertical and horizontal dimensions (chord length) of the DMD, with measurements recorded in millimeters by employing calipers. The authors proposed an algorithm named ‘HELP’ based on height-extent-length-pupillary involvement. These authors divided the cornea in three zone, with the help of AS-OCT and slit lamp examination: zone 1 (central 5 mm); zone 2 (paracentral 5 to 8 mm); zone 3 (> 8 mm). Patients with a DMD of less than 1.0 mm length and less than 100 μm in height in any zone underwent conservative management. For patients with DMD ranging from 1.0 to 2.0 mm in length and 100 to 300 μm in height in zone 1, with or without pupillary-axis involvement, surgical intervention was recommended. Eyes with similar-sized DMD detachments (1.0–2.0 mm in length and 100–300 μm in height) but located in zones 2 and 3 of the cornea, received conservative management. DMD exceeding 2.0 mm in length and 300 μm in height were managed differently depending on their location; if the detachment was in zones 1 and 2, patients underwent surgical intervention; if detachment was in zone 3, medical management was preferred [50]. Wylęgała and colleagues [20] examined 8 eyes with DMD after cataract surgery. The authors employed AS-OCT to assess the anterior segment and DM morphology. Using imaging, they classified the detachment as either localized (covering less than 1/3 of the corneal surface) or extensive (covering more than 1/3 of the corneal surface) [20]. Furthermore, they utilized the Mackool and Holtz [9] classification to categorize the presentation of DM as either planar or non-planar. Additionally, they evaluated the presence or absence of scrolling of the DM margins [20]. This categorization, primarily based on AS-OCT imaging, enabled the authors to determine the treatment approach. For patients with localized planar and extensive planar DMD, conservative management was initially pursued. In cases where conservative treatment failed after 2 weeks, the authors turned to surgical intervention, with the localization of the air injection site guided by AS-OCT [20]. A schematic representation of intracameral air injection is presented in Fig. 2. (Fig. 2).

SF6 is mentioned as a successful treatment option in several cases in the literature [19, 46, 51, 72]. Doi and colleagues [3] employed AS-OCT for both diagnosing and monitoring the treatment of DMD following cataract surgery in one patient. Due to pronounced corneal edema, conventional slit lamp examination provided insufficient clarity. Based on AS-OCT imaging the authors observed extensive DMD without scrolling and opted for a surgical approach with intracameral injection of SF6 gas, which led to complete resolution of the detachment within a one-month period [3].

Slit lamp examination is routinely carried out following cataract surgery, but AS-OCT is fundamental in patients with persistent corneal edema towards formulating a correct diagnosis of DMD and in tailoring successive management. Xie and colleagues reported a case of persistent corneal edema on slit lamp biomicroscopy of two months duration after phacoemulsification [23]. AS-OCT was performed and showed DMD along with multiple intra-membrane splits and an interlayer tear measuring 1.367 mm in length within the edematous corneal region [23]. Tractive forces between the split layers of DM were observed, with the most anterior layer exhibiting heightened reflectivity. The maximum depth of separation measured 394 μm, and the total width of DMD extended to 6.272 mm. The DMD had initially been treated at another facility as herpes simplex virus keratitis, without improvement. In this case, post-operative AS-OCT imaging facilitated the accurate diagnosis and corticosteroid eye drops with topical antibiotic coverage led to complete resolution of the detachment within a two-week time frame [23].

Zhou and colleagues [21] examined 4 eyes of 2 patients who were referred to their specialized cornea center due to persistent corneal edema post-cataract surgery. The authors performed AS-OCT, which enabled the detection of a previously undiagnosed DMD. In the first patient, AS-OCT imaging showed bilateral DMD. In the right eye imaging represented a retro-corneal line with heightened reflectivity and an inverted edge, whereas the left eye showed localized DMD with a planar configuration in proximity to the temporal paracentesis site. In the cases referenced [21, 23], patients experienced delays of up to two months post-surgery, receiving incorrect treatment due to difficulties in diagnosing DMD caused by corneal edema, which limited the effectiveness of slit-lamp examination. Immediate postoperative use of AS-OCT could have provided clear visualization despite the edema, enabling prompt detection of DMD and allowing for its timely management. In these instances, this approach would have significantly improved patient outcomes and their postoperative care by preventing complications associated with prolonged corneal edema and unnecessary topical medications on a cornea already compromised by recent intraocular surgery.

AS-OCT imaging enabled the tailoring of the treatment strategy based on the degree and morphology of DMD. The authors employed intracameral air injection in the right eye, and conservative therapy with topical corticosteroid eye drops and hyperosmotic agents in the left eye. This led to the complete resolution of DMD within a span of three weeks. In the second patient slit lamp examination showed diffuse corneal edema in both eyes [21]. AS-OCT enabled the identification of extensive DMD with a planar configuration measuring 2–3 mm in width and 0.36 and 0.40 mm from the posterior stroma. The authors opted for a surgical approach involving intracameral air tamponade in both eyes, achieving complete resolution in the right eye. However, owing to two treatment failures in the left eye, they switched to 14% C3F8 gas tamponade, resulting in the complete resolution of DMD. These cases highlight the efficacy of AS-OCT in guiding therapeutic decisions for post-cataract surgery DM-related complications [21]. C3F8 is mentioned as a successful treatment option by various authors [50, 63, 77, 86]. Venting incisions have also been used as an aid for pneumodescemetopexy under AS-OCT guidance to facilitate fluid drainage [42, 77]. Singh and colleagues described intraoperative SF6 gas tamponade guided by AS-OCT, which showed fluid accumulation between the stroma and the DMD. This observation prompted the authors to create venting incisions, which together with the gas tamponade effect, facilitated complete reattachment of the DM [77]. In the cases mentioned above [21, 42, 77], intraoperative AS-OCT proved to be valuable as a crucial asset in achieving accurate and effective treatment of DMD. By providing real-time visualization of the detachment and its surrounding structures, AS-OCT used intraoperatively during the DMD treatment [42, 77], enabled an accurate assessment of the extent and location of fluid accumulation. This allowed for a more tailored and safer approach in more complex cases of DMD, maximizing the chances of a successful reattachment.

**Fig. 3:**
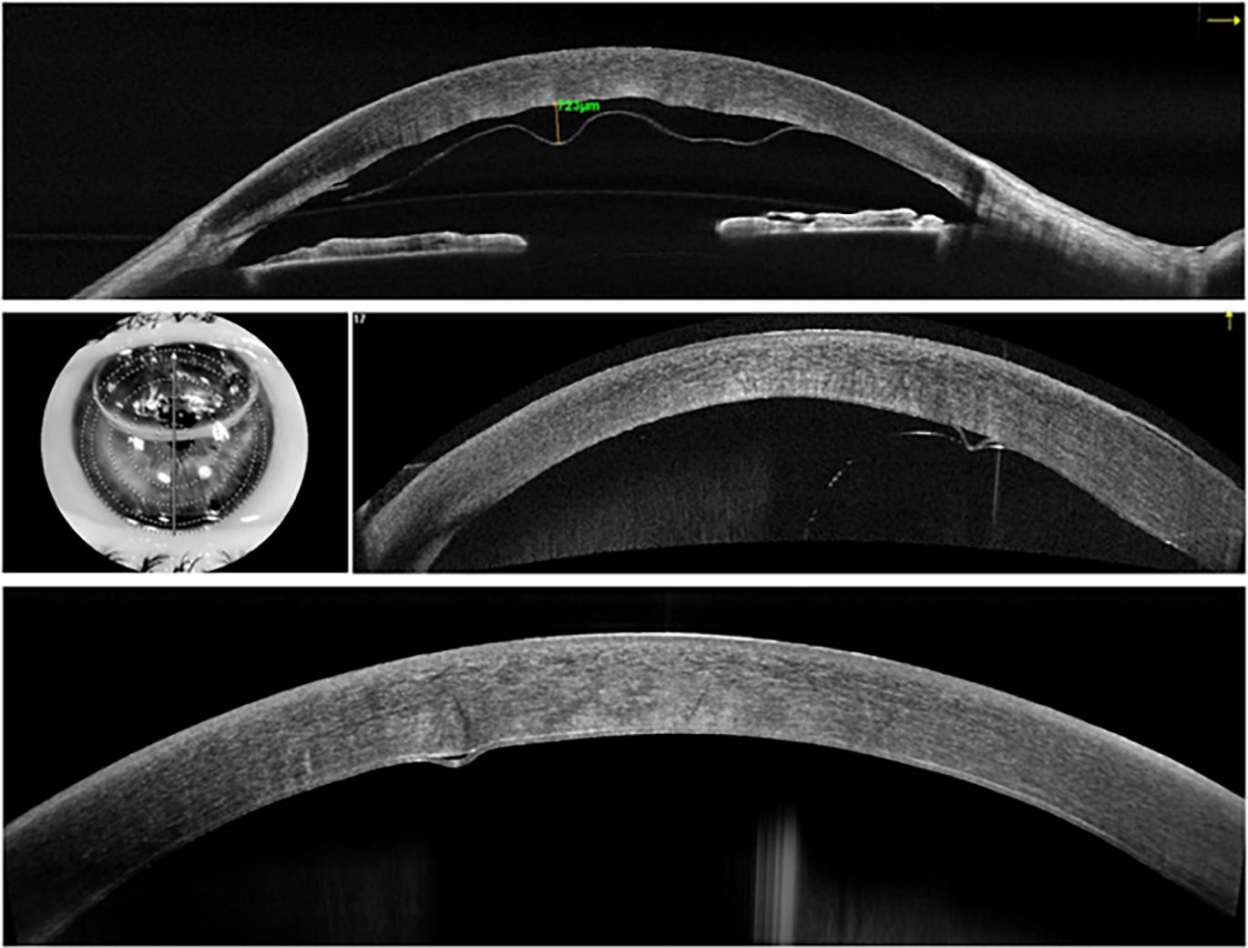
AS-OCT B-scans showing: **a** the postoperative Descemet’s Membrane Detachment (DMD) measuring 732 µm; **b** the AS-OCT scan depicting pneumodescemetopexy with an air bubble placed in the anterior chamber; **c** AS-OCT image of the reattached Descemet membrane 10 days post-treatment

In their retrospective case series, Li and colleagues [14] examined 12 eyes selected based on the persistence of central corneal edema for at least 3 days following cataract surgery. Post-operative DMD was classified with AS-OCT as planar or non-planar, including the assessment of associated scrolled margins [88]. Furthermore, topographic evaluation of the detachment (peripheral and/or central) was also carried out. Based on AS-OCT, a subclassification was created, including mild (peripheral involvement < 25%), moderate (peripheral involvement 25–50% of the cornea), and severe (> 50% or central involvement). The authors used air pneumodescemetopexy to manage DMD in all 12 patients, choosing the site of injection through AS-OCT imaging. Indeed, the site of air injection is fundamental in the treatment of DMD and AS-OCT enables to precisely visualize areas where the DM is detached or attached, in order to choose the latter as the precise injection point [14]. The utilization of AS-OCT in conjunction with traditional slit lamp examination expediates the diagnosis and treatment process in post-surgical DMD, leading to improved outcomes for patients. A further crucial factor is also the time elapsing between the occurrence of DMD following cataract surgery and its treatment [88] underscoring the vital role of AS-OCT in early diagnosis and intervention [14]. Figure 3 illustrates the sequential AS-OCT B-scans of the initial postoperative DMD, the pneumodescemtopexy, and the successful reattachment of the DM. (Fig. 3).

The appropriate timing for the use of AS-OCT in detecting DMD after cataract surgery varies, with a trend towards early postoperative assessment. Analysis of previous case reports reveals that AS-OCT is commonly performed within the first week post-surgery [3, 14, 19, 29, 78], with some instances where evaluations occur at two weeks to a month post-operation [21, 31, 42, 63]. The later use of AS-OCT, for instance after 2 months, could be linked to cases of delayed presentation or initial misdiagnosis [23]. Gharaee and colleagues [71] stated that post operative AS-OCT should be carried out whenever possible, it being an excellent non-invasive imaging modality for evaluating postoperative morphological features, wound integrity, and complications of the main incision. This suggests a preference for early postoperative imaging to facilitate timely management of DMD, while also accommodating cases where diagnosis is complicated by external factors or patient follow-up variations. Thus, potential AS-OCT integration in post-cataract surgery assessments could be justified and particularly beneficial when corneal edema is present and persists despite topical treatment. Under these circumstances, utilizing AS-OCT could provide the necessary detail to establish an accurate diagnosis, allow for precise visualization of the structures within the AC, and carry out subsequent tempestive treatment.

Overall, AS-OCT is a valuable tool in the management of DMD post-cataract surgery, enhancing the efficiency of diagnosis, which can be complex and not immediately apparent in cases of extensive corneal edema, but also in the topographic delineation of the detachment, facilitating informed treatment choices, and enabling close monitoring of therapeutic responses. AS-OCT imaging aids in identifying the injection point through the visualization of detachment topography and for aiding pneumodescemetopexy procedures. Postoperative treatment modalities range from conservative medical management to more aggressive surgical intervention, emphasizing the necessity for a tailored approach to DMD based on the extent of detachment, patient response to initial therapy, and anatomical considerations. The integration of AS-OCT in clinical practice, in postoperative cataract DMD, significantly contributes to the successful resolution of this complication, ultimately improving patient outcomes.

**Fig. 4:**
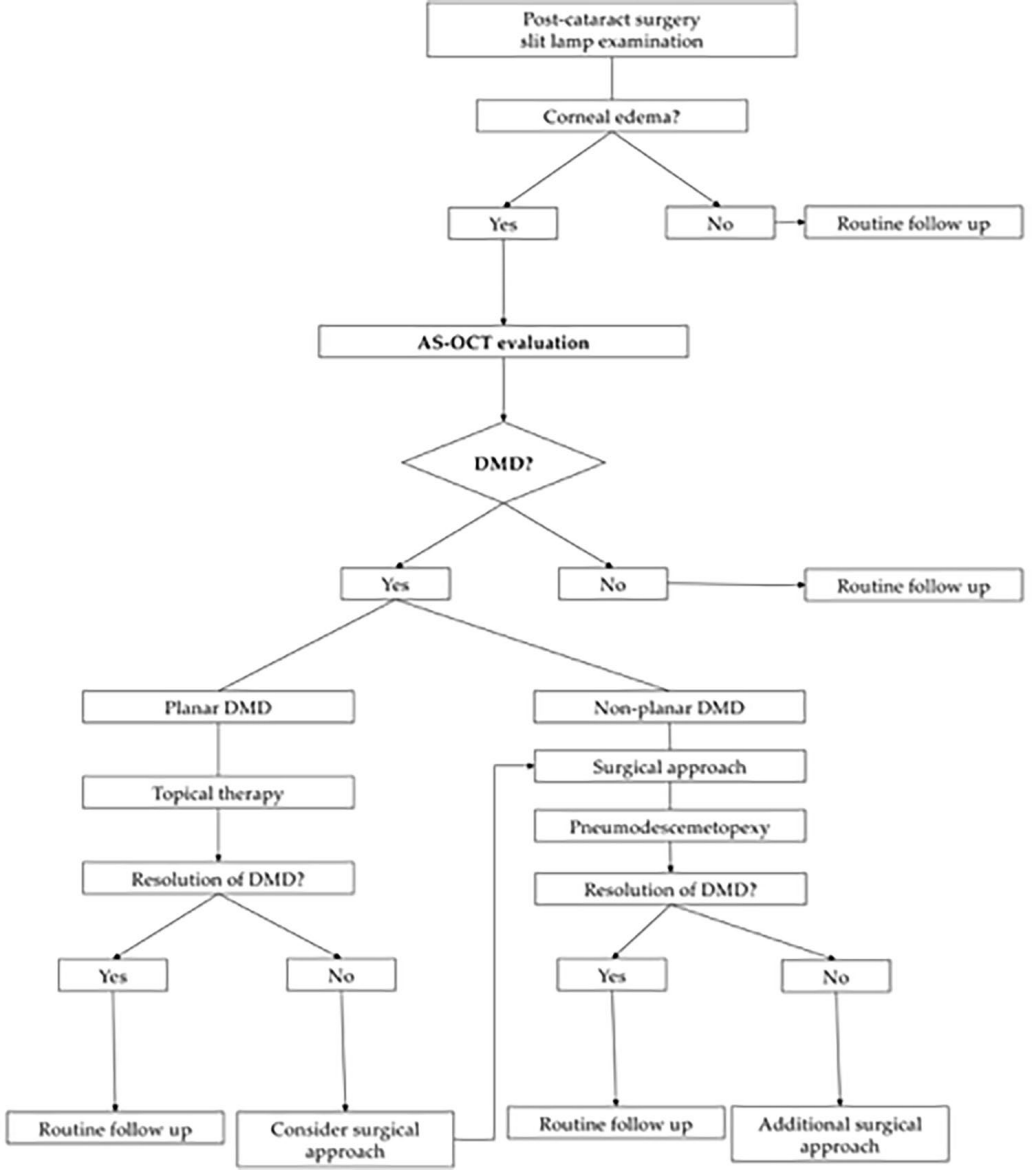
Diagnostic flowchart for Descemet membrane detachment (DMD) via anterior segment optical coherence tomography (AS-OCT)

AS-OCT should be used as soon as corneal edema appears after cataract surgery [3, 14, 19, 29, 71, 78], not only to help diagnose possible DMD but also to measure corneal thickness and anterior segment postoperative changes [71]. Using AS-OCT early allows for timely adjustments and to promptly pinpoint the cause of postoperative corneal edema in the management plan, helping to speed up recovery and reduce the risk of complications from delayed diagnosis [14, 19, 21, 23]. This approach supports better patient outcomes and can help avoid additional costs by reducing the need for further interventions [21, 23]. Figure 4 shows a diagnostic flowchart for DMD involving the use of AS-OCT as early as corneal edema manifests. (Fig. 4).

**Fig. 5:**
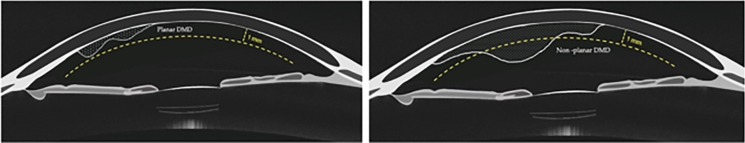
Schematic illustration of planar versus non-planar Descemet membrane detachment (DMD) classification as assessed via anterior segment optical coherence tomography

**Fig. 6:**
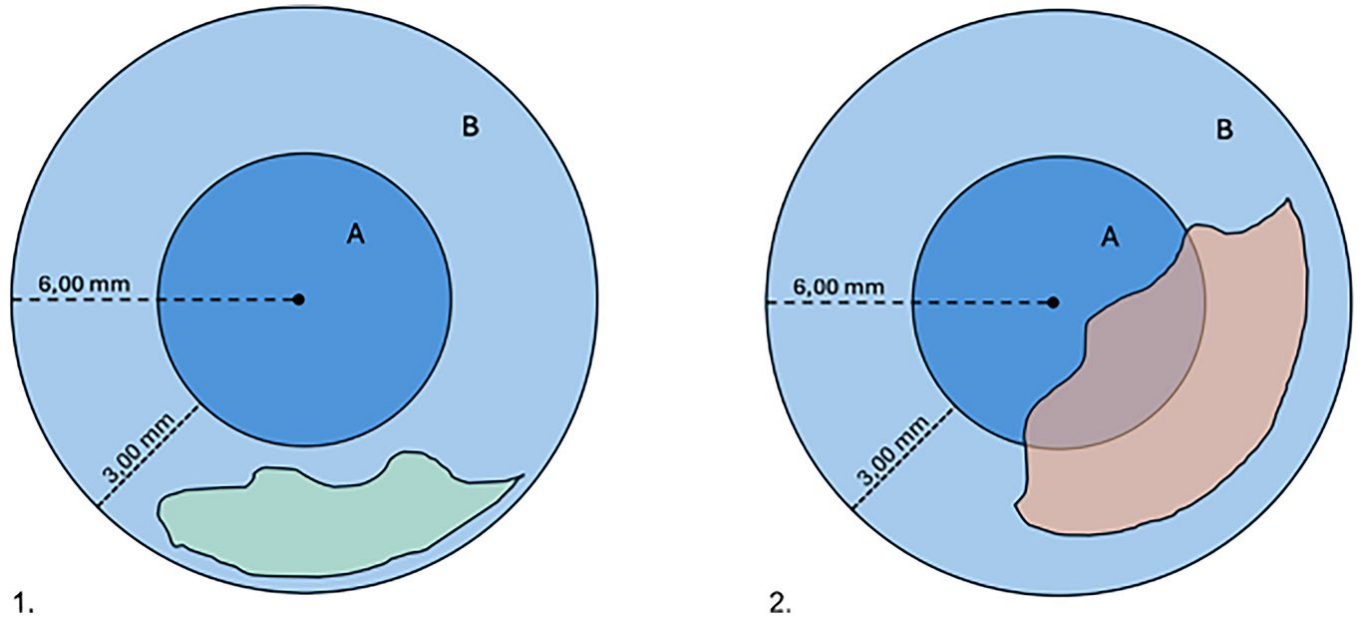
Schematic illustration showing the distinction between peripheral (1) and combined central-peripheral (2) Descemet membrane detachment (DMD)

Multiple studies [14, 19–21] have highlighted the critical role of AS-OCT in accurately defining and classifying DMDs as planar or non-planar based on the criteria established by Mackool and Holtz [9]. This classification represents a key diagnostic feature that is difficult to achieve with conventional slit-lamp biomicroscopy or other anterior segment imaging techniques. The classification of DMDs as planar or non-planar plays a key role in guiding the therapeutic approach, as extensively discussed and illustrated in the diagnostic flowchart. AS-OCT is instrumental in identifying and further refining this classification, as demonstrated in the cases reported by Li and colleagues [14], where AS-OCT determined the planarity of the DMD and differentiated between peripheral and combined central-peripheral detachments. Figures 5 and 6 provide schematic illustrations of planar versus non-planar DMD classification and the distinction between peripheral and combined central-peripheral DMD, respectively. (Figs. 5 and 6) (Table 2).

**Table 2:** Characteristics and management modalities of Descemet’s membrane detachment

Authors	Number of DMD	Type of AS-OCT	Role of AS-OCT	AS-OCT findings	Conservative treatment	Surgical treatment
Sharma et al. [19]	43	TD-OCT	Identify planar DMD	Mackool&Holtz classification	Complete resolution without therapy	None
		(Visante OCT 3.0; Carl Zeiss Meditec, Jena, Germany)	Measure extension of DMD	< 1 quadrant of the cornea		
Kumar et al. [50]	161	TD-OCT (Visante OCT; Carl Zeiss Meditec, Jena, Germany)	Assess dimension and localization of DMD	Vertical and horizontal measurement of DMD	< 1 mm in length, < 100 μm in height in any zone	1 < mm > 2 in length 100 < μm > 300 in height in zone 1
				Division of cornea in three zones	1 < mm > 2 in length 100 < μm > 300 in height in zone 2 and 3	> 2 mm in length, > 300 μm in height in zone 1 and 2
					> 2 mm in length, > 300 μm in height in zone 3	
Wylegala et al. [20]	8	TD-OCT	Morphology of anterior segment and DMD	Localization and extension of DMD	Localized/extensive and planar DMD	Localized/extensive and planar DMD with conservative treatment failure
		(Visante OCT; Carl Zeiss Meditec, Inc, Dublin, CA)	Local and extensive DMD	Mackool&Holtz holtz classification		Extensive non-planar with or without scrolling
				Scrolled edges		
Doi et al. [3]	1	SS-OCT (Casia SS-1000 OCT Tomey, Nagoya, Japan)	Diagnose and monitor DMD with corneal opacity	Localization and extension of DMD	None	Extensive DMD
				Scrolled edges		Severe visual impairment
Xie et al. [23]	1	SD-OCT (Cirrus; Carl Zeiss, Meditec, Inc., Dublin, CA)	Morphology of DMD	Localization and extension of the DMD	Spontaneous resolution with therapy	Not performed
			To Diagnose and monitor DMD with corneal opacity	Assessment of the presence of the schisis into DMD		
Zhou et al. [21]	4	TD-OCT (Visante OCT; Carl Zeiss, Meditec Inc., Dublin, Calif., USA)	Diagnose DMD in persistent corneal edema post-surgery	Mackool & Holtz classification	Localized and planar DMD	Scrolled edges
			Assess morphology	Localization and extension of DMD		Extensive DMD 2 < mm > 3 in width and < 1 mm in height
				c. Scrolled edges		
Li et al. [14]	12	SD-OCT (Topcon 3D OCT 1000 Mark II, Tokyo, Japan)	Diagnose and monitor DMD with corneal opacity	Mackool & Holtz classification	None	Planar DMD < 1 mm in height
			Morphology and localization of DMD	Scrolled edges		
				Topographic evaluation		
				Extension of DMD		

Descemet’s membrane detachment (DMD); Optical coherence tomography (OCT); Descemet membrane (DM); Time-Domain Optical coherence tomography (TD-OCT); Swept Source Optical coherence tomography (SS-OCT); Spectral domain Optical coherence tomography (SD-OCT)

## Artificial intelligence applications in AS-OCT for anterior segment disorders

In the continuously advancing landscape of diagnostic hardware and software, the integration of AI is transforming the approach to healthcare delivery. AI through machine learning (ML) and deep learning (DL) has made significant strides in the healthcare sector in many fields. The vast dataset of imaging available in ophthalmology, provides an ideal setting for the application of AI algorithms [89]. While AI in diabetic retinopathy and age-related macular degeneration has been groundbreaking through the analysis of vast datasets of OCT B-scans of the posterior pole, its application within corneal diseases is still an emerging frontier [90].

AS-OCT imaging dataset forms the foundation for implementing AI in addressing anterior segment conditions. AI shows promise in augmenting the analysis of corneal imaging, enhancing the precision and aiding the diagnosis of conditions such as keratoconus, infectious keratitis, and dry eye disease [90].

The intricate challenges posed by DMD after cataract surgery could benefit from the exploration of AI to offer prognostic and diagnostic guidance. Yousefi and colleagues developed an unsupervised ML to identify corneal conditions and predict the likelihood for corneal transplant based on AS-OCT features [90, 91]. Patefield and colleagues demonstrated the utilization of convoluted neural networks (CNNs), to predict which eyes may or may not have a graft detachment based on pre–DMEK AS-OCT images [90, 92]. The model proposed by Patefield and colleagues was found to have a better predictive level when compared with an experienced clinician who predicted based on clinical information and interpretation of AS-OCT images (sensitivity of 92% vs. 31%) [90, 92]. These studies have established a high degree of accuracy and sensitivity in comparison to traditional clinical assessment, showing the potential of AI to elevate the standard of patient care in corneal conditions. Several studies showed the potential of AI to improve surgical decision-making and enhance postoperative monitoring in corneal procedures such as keratoplasty and DMEK surgery [93, 94]. Heslinga and colleagues developed a CNN to accurately detect DMEK graft detachment, map its area and evaluate the detachment degree prompting tempestive treatment decisions. [90, 93] This type of integration could potentially be beneficial in correctly defining and delineating the area of DMD based on which treatment varies [20, 50, 88].

The integration of AI into clinical practice, however, is not without challenges. The laborious nature of data extraction and the potential biases within AI algorithms pose significant hurdles. In the future, if AS-OCT becomes routinely available in healthcare systems, similar to the vast integration of SDOCT in retinal and choroidal pathology, it could offer a potentially valuable tool in perioperative screening for anterior segment surgery including cataract surgery. The realization of this potential, however, necessitates further effort in research, cost–benefit analyses, and the development of real-life workflows in the routine clinical environment.

## Conclusions

In the context of cataract surgery DMD is a relatively rare complication. Until the advent of AS-OCT, diagnosis was made mainly through slit lamp evaluation but excessive corneal opacity can hinder early detection of DMD. High-resolution images provided by AS-OCT offer clarity in visualizing corneal anatomy, allowing clinicians to diagnose DMD and evaluate the extent and nature of the detachment with a precision that traditional methods may not provide, especially in cases of corneal edema.

Analysis of the current literature did not reveal significant correlations between the incidence of DMD and anatomical characteristics detectable by AS-OCT in the preoperative phase of cataract surgery, suggesting a limited role for this technology in preoperative risk stratification. Although intraoperative AS-OCT is not yet standard practice in cataract surgery due to cost considerations, its application could be of great value in the early detection and management of DMD. By providing real-time visualization of corneal structures during surgery, intraoperative AS-OCT allows for precise and prompt interventions, reducing the likelihood of postoperative complications. Additionally, AS-OCT remains extremely valuable in the postoperative phase, aiding in the accurate diagnosis of DMD, when other methods fall short, and supporting therapeutic decision-making, particularly in procedures such as pneumodescemetopexy where detailed topographic and morphological information of DMD is crucial to obtain a successful reattachment.

Ophthalmic surgical procedures are becoming increasingly data-driven and reliant on advanced imaging techniques and AS-OCT has a role in this trend by in enhancing clinical outcomes by improving diagnostic accuracy and early management ultimately increasing patient satisfaction and enhancement of the quality of care.

AS-OCT is relevant in the diagnosis of underlying causes in persistent postoperative corneal edema where topical treatment fails and towards successive targeted intervention.

AS-OCT is likely to become more widespread due to its ability to enhance intraoperative visualization of DMD. While DMD is a low-incidence complication of cataract surgery, there is an increasing drive to reduce any and all complications, especially for cataract surgery. The ability to detect and address DMD during the surgery itself with intraoperative AS-OCT allows for an immediate correction, sparing both the patient and surgeon from dealing with this issue postoperatively.

The potential integration of AI algorithms in AS-OCT analysis holds promise for enhancing diagnostic precision and therapeutic decision-making in cases of DMD, although further refinement and validation of AI models are necessary for widespread clinical implementation.

## Data Availability

No datasets were generated or analysed during the current study.
